# Strategies to improve the quality of life in patients with hereditary transthyretin amyloidosis (hATTR) and autonomic neuropathy

**DOI:** 10.1007/s10286-019-00624-w

**Published:** 2019-09-10

**Authors:** Thierry Gendre, Violaine Planté-Bordeneuve

**Affiliations:** 1 Department of Neurology, Henri Mondor University Hospital, 51 avenue de Lattre de Tassigny, 94010 Creteil Cedex, France; 2 Amyloid Network, Henri Mondor University Hospital, 51 avenue de Lattre de Tassigny, 94010 Créteil Cedex, France

**Keywords:** Hereditary transthyretin amyloidosis, quality of life, Autonomic neuropathy, Orthostatic hypotension

## Abstract

**Purpose:**

Hereditary transthyretin amyloidosis (hATTR) is a severe adult-onset progressive disease mainly involving the peripheral nervous system and the heart, with a prominent impact on the autonomic nervous system. This review summarizes the clinical aspects of autonomic dysfunction in hATTR, and their impact on quality of life as well as potential therapeutic options.

**Methods:**

Literature review.

**Results:**

Autonomic dysfunction, causing neurogenic orthostatic hypotension, gastroparesis, constipation, diarrhea, bladder dysfunction, and erectile dysfunction in males, has a major impact on the quality of life of patients with hATTR. Improvement of qualify of life in patients with hATTR implies periodic symptomatic screening and early management, taking into consideration comorbidities and medication side effects. The specific effect of the disease-modifying treatment on this aspect remains to be unraveled.

**Conclusions:**

Management of autonomic dysfunction in patients with hAATR is feasible and can result in improved qualify of life. Novel disease-modifying treatments for hAATR may contribute to improve autonomic dysfunction, although specific studies are required.

## Introduction

Hereditary transthyretin amyloidosis (hATTR) is a severe adult-onset progressive disorder characterized by tissue amyloid deposition of transthyretin [2, 18]. As the peripheral nerves and the heart are the main organs involved, the clinical picture is a length-dependent axonal neuropathy associated with cardiac conduction blocks or hypertrophic restrictive cardiomyopathy. At first, amyloid deposits alter small nerve fibers responsible for the impairment of thermalogic sensations associated with autonomic manifestations. Then, large nerve fibers will be altered, with impairment of other sensory modalities, unsteadiness and motor deficit.

Symptoms of autonomic dysfunction are a major feature of hATTR, as parasympathetic and sympathetic nerves are predominantly comprised of small fibers. Autonomic manifestations are pleomorphic, involving the cardiovascular, gastrointestinal and genitourinary systems. Their clinical expression may be misleading and often raise issues for the diagnosis. In the setting of a progressive sensory or sensorimotor neuropathy in a non-diabetic patient, they are an important clue for the diagnosis. Autonomic manifestations may be inaugural in approximately 10% of hATTR patients [18, 21]. Nowadays, the quality of life (QoL) is considered to be an important dimension in the management of patients and is often a surrogate endpoint in the recent trials using TTR stabilizers or RNA interference drugs [1, 3, 7, 8]. Due to the wide range of clinical expression, the autonomic symptoms are likely to have a major impact on the daily life of patients. However, little is known about the specific impact of autonomic dysfunction on the QoL of patients with hATTR.

In this paper, we describe the impact of autonomic manifestations on QoL in hATTR patients and report on the strategies to improve QoL through their management. Two case reports illustrate this important topic. To this aim, we gathered published data about autonomic dysfunction in hATTR and in other diseases, as well as describe our own clinical experience.

## Case vignettes

### Case 1

A 45-year-old woman of Portuguese origin has a 10-year follow-up of hATTR-Val30Met polyneuropathy. Initially, she presented intermittent diarrhea along with a progressive weight loss of 10 kg. Gastrointestinal investigations including a coloscopy with biopsy remained negative. Two years later, she complained of progressive ascending symmetrical paresthesia in her lower limbs and suffered from dizziness when she stood up. At that time, she was able to walk unaided. Neurological examination showed loss of temperature and pain sensations in a stocking and gloves distribution; muscle strength was preserved. In addition to intermittent postprandial diarrheas, autonomic manifestations included mild orthostatic hypotension with blurred vision. The diagnosis of hATTR was set on the clinical picture in the context of positive family history in her father, the identification of Val30Met-ATTR variant on the genetic test and of amyloid deposits on a salivary gland biopsy. During the diagnostic work-up, a pacemaker was implanted because of heavy cardiac conduction blocks. The patient refused liver transplantation. She then received a TTR stabilizer orally.

During the course, her sensorimotor condition slowly deteriorated. So far, she walks with one stick and two foot-orthosis. Autonomic manifestations are also progressing. These include daily explosive diarrhea with episodes of fecal incontinence, early satiety and nausea due to gastroparesis. Also, she reports malaise, blurred vision or fatigue when standing up, and presented several traumatic falls. On examination, severe orthostatic hypotension was elicited. Lastly, she presented repeated episodes of urinary tract infection with dysuria. A bladder scan detected a residual volume of 300 ml after micturition symptomatic of chronic urinary retention. Renal and urinary tract echography were normal along with the glomerular filtration rate. The management of autonomic manifestations included dietary changes, reducing the amount and increasing the frequency of meals, intermittent oral antibiotics for bacterial overgrowth-related diarrhea, and loperamide or racecadotril for diarrhea. The treatment of orthostatic hypotension required elastic stockings and midodrine 25 mg/day. Urinary intermittent self-catheterization was performed 5 times a day. Such management led to improving her social and familial relationships along with her ability to perform activities of daily living.

### Case 2

A 70-year-old woman of Algerian origin has a 3-year history of hATTR-Thr69Ile polyneuropathy and cardiomyopathy. For the past 2 years, she has complained of ascending symmetrical paresthesia in her lower limbs. Meanwhile, she had intermittent episodes of diarrhea, recurrent nausea, and vomiting, associated with a severe weight loss of 22 kg. Within 6 months, she presented three syncope and increasing dyspnea. Neurologic examination showed a patient in poor general condition, walking unaided with normal strength, mild temperature and pain sensory loss in a stocking and gloves distribution, and areflexia in the lower limbs. Most of the time, she was unable to stand up because of severe orthostatic hypotension. Neurophysiological tests displayed a sensory axonal neuropathy in all four limbs and severe alteration of the heart rate variability to a deep breathing test. The diagnosis of hATTR was confirmed, based on the sensory-autonomic neuropathy associated with hypertrophic restrictive cardiomyopathy on echography, identification of the Thr69Ile-ATTR variant on the genetic test and amyloid deposits on a salivary gland biopsy. During the work-up, hypertrophic restrictive cardiomyopathy, cardiac conduction blocks and arrhythmias were documented. A cardioverter was implanted, and an oral TTR stabilizer was initiated. During the following year, the patient condition worsened. She was recently admitted for orthopnea, edema associated with recurrent vomiting episodes and diarrhea. Cardiac failure was diagnosed on the top of the gastrointestinal dysautonomia. Cardiac symptoms regressed after the administration of diuretics. However, orthostatic hypotension dramatically exacerbated, preventing the patient from standing up. Midodrine was increased progressively to 25 mg/day with partial response. Vomiting improved gradually after intravenous administration of erythromycin. Also, the introduction of octreotide decreased the diarrheic episodes. The patient could be discharged home but still remained dependent for all daily life activities with a poor QoL due to the autonomic dysfunction.

## Identifying autonomic involvement in TTR amyloidosis

A careful clinical approach is the most useful way to identify possible autonomic dysfunction. The screening of diverse symptoms must be systematic, covering the different aspects of autonomic dysfunction (Table 1). This section summarizes the approaches to detect autonomic dysfunction in hATTR.

**Table 1:** Evaluation of autonomic dysfunction

Autonomic involvement	Symptoms	Investigations
Cardiovascular		
Arrhythmia	Palpitations	Holter-ECG, HRDB
Orthostatic hypotension	Postural fatigue, dizziness, blurred vision, falls, syncope	Fall of systolic BP of 30 mmHg or diastolic BP of 10 mmHg within 3 min of standing up
Gastrointestinal		
Gastroparesis	Early satiety, slow digestion, post-prandial nausea and vomiting, anorexia	None
Low gastrointestinal	Constipation, diarrhea, alternating diarrhea and constipation, weight loss	Albumin serum level, body mass index
Urinary		
Parasympathetic	Dysuria, incomplete bladder emptying, urinary tract infections, overflow incontinence, urinary retentions	Urodynamic tests, creatinine serum level, renal ultrasound
Sympathetic	Pollakiuria, stress nature incontinence	Urodynamic tests
Sexual	Erectile dysfunction	None

*BP* blood pressure, *ECG* electrocardiogram, *HRDB* heart rate response to deep breathing

Cardiovascular autonomic neuropathy is the most threatening autonomic manifestation, as it poses a risk of sudden death. It usually starts with resting tachycardia or arrhythmia. The heart rate might fluctuate but does not exhibit normal variations in response to changing physiological situations, such as Valsalva maneuver or deep breathing. These abnormalities can be detected on electrocardiogram during deep breathing. Meantime, baroreflex dysfunction causes impaired blood pressure regulation, resulting in neurogenic orthostatic hypotension. Radiolabeled meta-iodobenzylguanidine scintigraphy is also a reliable diagnostic method to screen for cardiac sympathetic denervation, even in the early stages of the disease [17].

Orthostatic hypotension (a sustained drop of at least 20 mmHg in systolic or 10 mmHg in diastolic blood pressure within 3 min of standing up or head-up tilt) is the most disabling sign of cardiovascular autonomic dysfunction [12]. Orthostatic hypotension can be symptomatic or asymptomatic. Symptoms are a consequence of organ hypoperfusion, and include dizziness, lightheadedness, blurred vision when standing, falls and syncope. Importantly, orthostatic hypotension can be aggravated by recurrent episodes of diarrhea or by medications administrated concomitantly for fluctuating hypertension, heart failure, or neuropathic pain.

Gastrointestinal autonomic symptoms are diverse and frequent [26]. They are often difficult to distinguish from the direct consequences of amyloid deposition in the gastrointestinal tract. Gastroparesis is initially characterized by early satiety, preventing normal meals, or slow digestion. Then, postprandial nausea occurs leading to anorexia. Subsequently, it manifests as uncontrollable recurrent postprandial vomiting episodes leading to dehydration, food restriction, severe weight loss, malnutrition, and vitamin deficiency. Several methods are available to assess gastric emptying, such as radioactively-marked meals scintigraphy or gastroscopy.

hATTR patients also present lower intestinal tract disturbances, including severe constipation or recurrent postprandial explosive diarrhea, but frequently alternating of both. Sometimes, the diarrhea is largely predominant and leads to fecal incontinence. Small bowel motility can be investigated with ambulatory 24-h manometry.

Bladder dysfunction is also frequent. Lower urinary tract dysfunction is characterized by damage to both sacral parasympathetic fibers and motor sympathetic and somatic nerves [13]. Incomplete bladder emptying increases the risk of urinary tract infections. Ultimately, the underactive bladder leads to a large residual volume after micturition, urinary retention and overflow incontinence.

Erectile dysfunction in males is an early manifestation of the disease, and may predate the onset of sensory neuropathy. Retrograde ejaculation is frequent in patients with an atonic bladder.

Abnormal pupillary responses, such as miosis and light near dissociation, may be observed along with scalloping pupils. These typically cause little or no symptoms.

There are available validated scales to screen for symptoms of autonomic dysfunction. The COMPASS-31 [22] is a comprehensive questionnaire including 101 items, used in the phase-3 trials with patisiran, a small interference RNA antiTTR [1]. The Compound Autonomic Disability Test questionnaire [11] is a simpler tool, which allows the screening of the major components of autonomic neuropathy in daily practice. However, its ability to quantify the impact of dysautonomia has not been assessed. Also, the Kumamoto clinical score [24], developed in Japan, has been proposed to assess autonomic symptoms, sensorimotor abnormalities, and visceral organ impairments. However, it has not been widely tested.

## Impact of autonomic dysfunction on the QoL

The impact of autonomic dysfunction on the QoL of patients with hATTR remains understudied. In other chronic diseases with involvement in the autonomic nervous system, like diabetes mellitus, or rare disorders such as familial dysautonomia (Riley–Day syndrome, hereditary sensory and autonomic neuropathy type 3), the burden of autonomic dysfunction has been well quantified using the SF-36 questionnaire [15, 20].

In patients with hATTR and polyneuropathy, the impact of autonomic dysfunction on QoL is presumably extremely important. In Portuguese Val30Met patients, autonomic symptoms altered the Norfolk QOL-DN at an early stage of the disease, even in the absence of walking disability or impairment of daily living activities [9, 25].

Cardiovascular autonomic dysfunction, in particular, orthostatic hypotension, results to significant disability. In early stages, patients complain of dizziness, which mainly impacts on their daily motion. In more severe cases, patients are unable to stand up and become progressively wheelchair-bound and bedridden. Moreover, syncope is associated with traumatic injuries [16].

Gastrointestinal dysfunction has a major impact on the QoL of patients with hATTR [26]. Constipation induces discomfort and pain. On the other hand, diarrhea badly interferes in the daily activities and therefore on the professional, social familial life of patients. Explosive diarrhea and fecal incontinence are particularly deleterious in this setting. Upper gastrointestinal manifestations including early satiety, nausea and/or vomiting can be permanent with similar consequence on the patient life. Their combination with chronic or alternating diarrhea readily triggers dehydration and worsening of orthostatic hypotension. Overall, gastrointestinal manifestations alter the nutritional status leading to severe weight loss, food restriction, and malabsorption of essential nutrients and vitamin deficiency. Early occurrence of gastrointestinal manifestations, diarrhea, and malnutrition impact the patient survival [23].

The neurogenic lower urinary tract manifestations particularly incontinence interferes with work, social life, daily activities of patients with hATTR associated with a degrading image. Incomplete bladder emptying facilitates urinary tract infections, sepsis, and may negatively impact survival [10]. Moreover, erectile dysfunction affects both the emotional and sexual life of patients and their spouses.

**Fig. 1:**
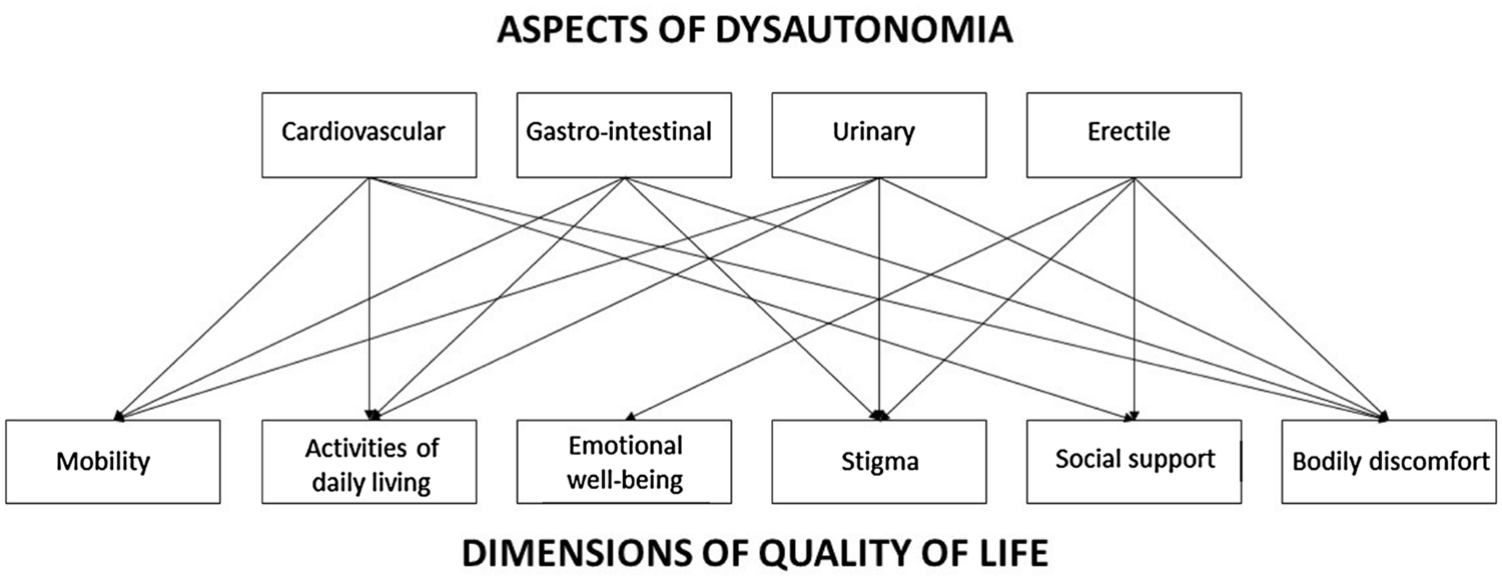
Impact of specific aspects of autonomic dysfunction on dimensions of QoL in patients with hereditary transthyretin amyloidosis

Thus, each aspect of dysautonomia deteriorates the QoL of patients with hATTR in different ways. Taken together, they heavily impact the patients’ life, in particular, social, familial dimensions as well as working abilities (Fig. 1). In addition, they significantly impact life expectancy.

## Management of autonomic dysfunction in hAATR

Appropriate management of autonomic dysfunction is key to improving the QoL of patients with hATTR. Symptomatic treatment should address carefully all aspects of autonomic dysfunction (Table 2). Both non-pharmacological measures and pharmacological treatments should be considered. In more severe cases, the presence of co-morbidities and concurrent autonomic symptoms is often difficult disentangle. On the one hand, one autonomic manifestation may aggravate the other, such as severe diarrhea worsening orthostatic hypotension. On the other hand, symptomatic treatment may impact negatively other function. For instance, volume expansion induced by fludrocortisone, a common pharmacological treatment for orthostatic hypotension, can result in increased pre-load and worsening heart failure. Arrhythmias can be a presenting feature of cardiovascular autonomic dysfunction. The identification of ventricular arrhythmia may require the implantation of a cardioverter defibrillator [14].

**Table 2:** Management of autonomic dysfunction in hATTR

Autonomic involvement	Non-pharmacological measures	Pharmacological approaches
Cardiovascular dysfunction		
Arrhythmia	Implantable cardioverter defibrillator	
Orthostatic hypotension	Medications review, elastic stockings, head-up position when sleeping, progressive standing up, salt intake	Midodrine 2.5–40 mg/day Fludrocortisone 0.05–0.2 mg/day Droxidopa 300–1800 mg/day
Gastrointestinal dysfunction		
Weight loss	Oral nutritional supplements	Vitamin supplementation, cholestyramine 12 g daily, parenteral nutrition
Gastroparesis	Frequent, smaller meals	Dopamine receptor antagonists, Motilin receptor agonists
Constipation	Increased fiber intake	PEG, sodium picosulfate
Alternating diarrhea and constipation		Antibiotics
Diarrhea	Stoma surgery	Loperamide 2–16 mg daily, Octreotide 100 µg daily
Bladder dysfunction		
Parasympathetic	No drink at bedtime, intermittent catheterization	α-blockers
Sympathetic	No drink at bedtime	Muscarinic receptor agonist, Mirabegron
Erectile dysfunction	Vacuum constriction devices, penile prosthesis	Phosphodiesterase type 5-inhibitors, intracavernous injections, intraurethral alprostadil

*PDE5-I* phosphodiesterase type 5 inhibitor, *PEG* polyethylene glycol

The management of orthostatic hypotension is discussed in depth elsewhere in this issue of Clinical Autonomic Research. In brief, non-pharmacological measures such as compression garments (i.e., compression stockings), elevation of the head 30°–45° when sleeping, and progressive position changes (e.g., when standing up) are the first line recommendations. Liberalization of water and salt intake is also recommended, except in those cases that may aggravate heart failure in patients with severe cardiopathy. Adjustment of cardiac medications might be necessary (e.g., reduction of diuretic dose), along with correction of aggravating factors such as diarrhea. If correction of aggravating factors and non-pharmacological measures are insufficient, pharmacological treatment is required. Midodrine, a short-acting alpha1-adrenoreceptor agonist, can increase blood pressure and improve symptoms of orthostatic hypotension. Dosages range from 2.5 to 40 mg/day. Fludrocortisone is longer acting, and induces salt and volume retention. Dosages range from 0.05 to 0.2 mg/day. As mentioned, it carries the risk of decompensating underlying cardiomyopathy. Both drugs can cause or aggravate hypertension in the supine position. Droxidopa (Northera^®^) is approved in the U.S. and some countries in Asia, and is safe and effective for treating symptomatic neurogenic orthostatic hypotension in patients with hATTR.

The management of gastrointestinal symptoms is challenging [27], and is discussed in depth elsewhere in this issue of *Clinical Autonomic Research*. In brief, to improve gastroparesis, patients should eat more frequent, smaller meals, and adjust their diet. The dopamine receptor antagonists, metoclopramide or domperidone, are the initial therapeutic option. In the case of refractory vomiting, intravenous therapy along with IV fluids might be required. If unsuccessful, motilin receptor agonists (erythromycin, clarithromycin, and azithromycin) can be added to promote gastric emptying. Constipation requires dietary changes such as increased fiber intake. Osmotic-acting laxatives (polyethylene glycol 13–80 mg daily) are useful. Lactulose is another alternative. In severe cases, a rectal enema may be necessary. Alternating constipation and diarrhea may be caused by intestinal bacterial overgrowth. Antibiotics (ciprofloxacin, tetracycline, metronidazole, rifaximin) for 5–7 days can be used as soon as the patient feels increased bowel activity before diarrhea. Some patients require frequent courses of antibiotics given every 3–4 weeks. Chronic diarrhea frequently induces malabsorption. In addition to diet modification reducing fiber intake, loperamide is the first option (2–16 mg orally daily). Also, the treatment of bile acid malabsorption by bile acid sequestrants (cholestyramine 4 g before meals) may be effective in combination with a fat-reduced diet [27]. Octreotide, a somatostatin analog, (50 µg subcutaneous twice daily) can be used in patients with intestinal pseudo-obstruction [6].

The nutritional status of hATTR patients with autonomic symptoms should be evaluated. In this setting, the albumin and modified body mass index are useful tools and have been correlated with survival [23]. Vitamin deficiencies will be supplemented at first by the intravenous route eventually associated with oral administration of nutritional supplements. Parenteral nutrition may be required to improve the nutritional status in severe cases [19]. In this setting, the guidance from a dietician with expertise on hATTR may be necessary.

The management of bladder dysfunction in patients with hTTRA is discussed in depth elsewhere in this issue of *Clinical Autonomic Research*. In brief, a urodynamic study is helpful to evaluate parasympathetic and sympathetic dysfunction [4]. In bladder retention, micturition is recommended at a regular interval, while liquid intake should be reduced before nighttime. Treatment with α-blockers (e.g., tamsulosin) can dilate the urethra and promote urination, but they are not routinely recommended as they consistently cause or aggravate orthostatic hypotension. Intermittent catheterization can be necessary in case of high post-void residual volume (e.g., above 100 ml), eventually contributing to urinary tract infections. In case of urgency due to overflow incontinence, muscarinic receptor antagonists (e.g., oxybvutinin, trospium) may be used, although they can have side effects, including cognitive impairment. Mirabegron, a beta-3-adrenergic agonist, can relax the detrusor without cognitive or hypotensive side effects. In many cases, combining a detrusor relaxant with scheduled intermittent self-catheterization is required.

Erectile dysfunction can be successfully treated with phosphodiesterase type 5-inhibitors (sildenafil), although they consistently cause orthostatic hypotension. Patients should be advised to avoid the standing position during and several hours after using these drugs. In all cases, a cardiac evaluation including blood pressure supine and standing is recommended before starting erectile dysfunction pharmacolotical treatments. Other potential options are intracavernous injections, intraurethral alprostadil, and vacuum constriction devices.

The best approach to preserving autonomic-related QoL in patients with hATTR would be to prevent further neurodegeneration of autonomic pathways as early as possible with disease-modifying therapeutics. However, the impact of the recently developed disease-modifying treatments on the autonomic neuropathy remains poorly understood. The effect of liver transplantation on autonomic symptoms has been controversial. In the long term, autonomic symptoms remain stable after liver transplantation in early-onset ATTR-Val30Met patients. In other subsets of hATTR patients, they often deteriorate progressively after liver transplantation along with the sensorimotor neuropathy [5, 28]. Regarding TTR stabilizers, the effect of tafamidis on autonomic symptoms remains poorly studied. The effect of tafamidis on autonomic dysfunction in hATTR is reviewed in depth elsewhere in this issue of *Clinical Autonomic Research*. Among the recently approved gene-modifying therapies, the small interference RNA agent, patisiran, has shown to significantly improve the autonomic symptoms measured by the COMPASS-31, whereas it was not specifically studied in the NEURO-TTR trial on the antisense anti-TTR oligonucleotide, inotersen. The impact of patisiran and inotersen on autonomic dysfunction in patients with hAATR is reviewed in depth elsewhere in this issue of *Clinical Autonomic Research*.

## Conclusions

Symptoms of autonomic dysfunction have a major impact on the QoL and survival of patients with hATTR. However, only a few studies are available to quantify this aspect of the disease. A careful and systematic approach to each aspect of autonomic dysfunction is key to improving the QoL of patients with hATTR. Management is often challenging and requires skilled providers often in a multidisciplinary team. Novel disease-modifying treatments for hAATR may contribute to improve autonomic dysfunction, although specific studies are required.
